# Rapid response to dupilumab in an adult patient with eosinophilic esophagitis and allergic asthma 

**DOI:** 10.5414/ALX02485E

**Published:** 2024-04-05

**Authors:** Benjamin Klein, Regina Treudler

**Affiliations:** 1 Leipzig Comprehensive Allergy Center LICA-CAC, Department of Dermatology, Venereology and Allergology, University of Leipzig, Leipzig, Germany,; 2 Division of Rheumatology, Department of Internal Medicine, University of Michigan, Ann Arbor, MI, USA, and; 3 Institute of Allergology, Charité Universitätsmedizin Berlin, Berlin, Germany

**Keywords:** eosinophilic, esophagitis

## Abstract

Abstract. Background: Eosinophilic esophagitis (EoE) is an inflammatory disease of the esophagus that belongs to the spectrum of Th2-mediated diseases. It is often associated with atopic comorbidities such as allergic asthma (AA) and poses a therapeutic challenge. Case report: We report on a 43-year-old patient with EoE and AA who did not show sufficient therapeutic control despite standard therapy. We started treatment with dupilumab, whereupon both EoE and AA rapidly improved and complete symptom resolution could be documented. The response to dupilumab was assessed by laboratory monitoring and gastroscopy, which showed a reduction of markers of type II inflammation and eosinophilic infiltrates in the esophagus. Summary: Our report emphasizes the effective and safe use of dupilumab as a treatment option for EoE with concomitant beneficial effects on AA.

## Introduction 

Eosinophilic esophagitis (EoE) characterizes an eosinophilic inflammation in the esophagus, leading to dysphagia and episodes of food-induced immediate response of the esophagus (FIRE) [1]. Dupilumab, which is approved for moderate to severe allergic asthma (AA), chronic rhinosinusitis, atopic dermatitis and, recently, for EoE, is a promising drug for the treatment of different type 2 inflammatory diseases [2, 3, 4]. Here, we describe a patient with marked type 2 inflammation, consisting of EoE and AA, who showed an excellent therapeutic response to dupilumab. 

## Case report 

**Figure 1:**
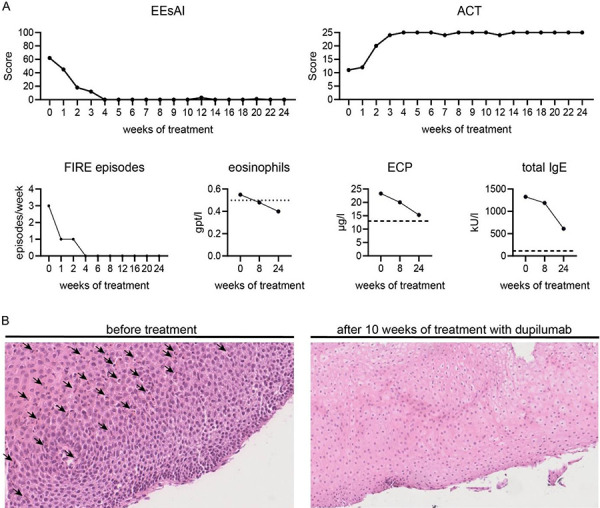
Assessment of clinical and serological response to dupilumab. A: Eosinophilic Esophagitis Activity Index (EEsAI), Asthma Control Test (ACT), and food-induced immediate response of the esophagus (FIRE) episodes were measured up to 24 weeks after initiation of dupilumab. Laboratory tests were performed 8 and 24 weeks after first dose of dupilumab. B: Hematoxylin and eosin staining of esophagus biopsies obtained during gastroscopies before (left) and 10 weeks after (right) treatment with dupilumab. Arrows indicate eosinophils in esophageal mucosa.

A 43-year-old patient presented to our allergy department with symptoms of histologically confirmed EoE with strong dysphagia over 2 years. Previous treatments consisted of pantoprazole 40 mg once daily and budesonide 1 mg as melt tablet. Under this regimen he showed inadequate disease control with an Eosinophilic Esophagitis Activity Index (EEsAI) of 62/100 and esophageal Candidiasis requiring amphotericin B lozenges (Figure 1A). EoE led to avoidance behaviors of eating in restaurants and dieting with consecutive weight loss. The patient reported ~ 2 – 3 FIRE episodes per week with a wide variety of foods, especially corn (Figure 1A). He suffered from AA and allergic rhinitis (AR) with onset in early childhood, which was treated with continuous inhaled corticosteroids (fluticasone propionate 125 µg 1-0-1) and inhaled bronchodilators (salbutamol) as needed. The patient showed exacerbations of AA and AR during cold wet weather, and his AA was not controlled during his first visit with an Asthma Control Test (ACT) of 11/24 (Figure 1A). 

Complete blood count (CBC) revealed blood eosinophilia of 7.3% and 0.55 gpt/L (normal range < 0.5 gpt/L), and laboratory tests showed elevated eosinophil cationic protein (ECP) of 23.3 µg/L (< 13 µg/L) and elevated total IgE of 1,327 kU/L (< 114 kU/L) (Figure 1A). Furthermore, there was a polyvalent type 1 sensitization to respiratory allergens (birch, lichen grass, dust mite, *Alternaria*), as well as food allergens (peanut, egg-white) without previous anaphylactic reactions (Table 1). A gastroscopy had shown the onset of esophageal sparing and histologic accumulation of eosinophils in the mucosa (> 20 eosinophils/HPF), indicative of EoE (Figure 1B). 

Since the patient had multiple type 2 inflammatory diseases with inadequate response and side effects, we used an individual approach with dupilumab (Sanofi-Regeneron, Germany) 300 mg every 2 weeks subcutaneously. During therapy, weekly disease activity scores (EEsAI and ACT) and laboratory controls documented rapid clinical improvement (Figure 1A). 

After just 2 weeks, the patient showed a clear response in the activity scores, which increasingly improved to their maximal value after 4 weeks and remained stable over the next 32 weeks (Figure 1A). Strikingly, previous FIRE-causing products (e.g., corn) could be eaten without problems, and FIRE episodes were completely absent 4 weeks after dupilumab initiation (Figure 1A). Furthermore, the patient presented an ACT of 24/24 (complete control) (Figure 1A). Noteworthy was a reduction of eosinophils to normal range after 8 weeks, a reduction of ECP, and a strong reduction of total IgE after 24 weeks, which is consistent with previous reports (Figure 1A) [5]. Control gastroscopy after 10 weeks of treatment showed no evidence of EoE, and histologically no accumulations of eosinophils were detectable (Figure 1C). Over time, the inhaled asthma medications could be de-escalated to an on-demand treatment and the patient was able to stop long-term therapy with pantoprazole and budesonide melt tablet. Under ongoing dupilumab therapy he is asymptomatic regarding EoE and bronchial asthma after 48 weeks of follow-up. 

## Discussion 

According to the information for healthcare professionals, dupilumab is indicated for the treatment of EoE in adults and adolescents aged 12 years and over with a body weight of at least 40 kg who are inadequately treated with conventional drug therapy, who cannot tolerate it, or for whom such therapy is not an option. Unfortunately, an additional benefit was not recognized by the Federal Joint Committee (german: Gemeinsamer Bundesausschuss, G-BA) [6]. 

Of note in our patient is the rapid effect of dupilumab after 2 weeks and the complete resolution of symptoms after 4 weeks with regard to EoE including FIRE episodes and allergic asthma. While dupilumab was administered once weekly at a dose of 300 mg in previous studies [2], the patient’s health insurance company agreed to only 1 dose every 2 weeks, and he had a positive effect even with this extended injection interval. 

In conclusion, our report highlights the effective and safe use of dupilumab as a treatment option in EoE [7] with concomitant beneficial effects on asthma. 

## Authors’ contributions 

BK collected the data, BK and RT conceived and designed the analysis. BK wrote the manuscript. RT supervised the manuscript. 

## Funding 

BK received no funding for this work. 

## Conflict of interest 

R. Treudler has received honoraria for lectures and/or consulting and/or conference support from Sanofi-Genzyme, ALK-Abello, Takeda, Novartis, Hautnetz Leipzig, Fraunhofer-IZI Leipzig, AbbVie, Pfizer, CSL Behring, LeoPharma, all unrelated to this paper. 

B. Klein declares that he has no conflict of interest. 

**Table 1.** Skin prick test and specific IgE in peripheral blood.

**Prick test substance**	**Wheal (in mm)**	**Flare**
NaCl	0	0
Grass mix	5	> 20
Rye	4	15
Birch	4	> 20
Hazel	4	> 20
Dust mite	4	15
Alternaria	3	15
Dog	3	> 20
Cat	9	> 20
**Specific IgE**	**Value (in kU/L)**	**CAP classification**
rBet v 1	30.1	CAP 4
rPhl p 1/5	15.00	CAP 3
D. pteronyssinus	1.52	CAP 2
D. farinae	2.52	CAP 2
Alternaria	14.52	CAP 3
